# The Combination of Mild Salinity Conditions and Exogenously Applied Phenolics Modulates Functional Traits in Lettuce

**DOI:** 10.3390/plants10071457

**Published:** 2021-07-16

**Authors:** Leilei Zhang, Erika Martinelli, Biancamaria Senizza, Begoña Miras-Moreno, Evren Yildiztugay, Busra Arikan, Fevzi Elbasan, Gunes Ak, Melike Balci, Gokhan Zengin, Youssef Rouphael, Luigi Lucini

**Affiliations:** 1 Department for Sustainable Food Process, Università Cattolica del Sacro Cuore, 29122 Piacenza, Italy; leilei.zhang@unicatt.it (L.Z.); erika.martinelli1@unicatt.it (E.M.); biancamaria.senizza@unicatt.it (B.S.); mariabegona.mirasmoreno@unicatt.it (B.M.-M.); luigi.lucini@unicatt.it (L.L.); 2 Department of Biotechnology, Faculty of Science, Selcuk University, Selcuklu, Konya 42130, Turkey; eytugay@selcuk.edu.tr (E.Y.); busra.arikan@selcuk.edu.tr (B.A.); fevzi.elba@gmail.com (F.E.); melikebalci9618@gmail.com (M.B.); 3 Physiology and Biochemistry Research Laboratory, Department of Biology, Science Faculty, Selcuk University, Selcuklu, Konya 42130, Turkey; akguneselcuk@gmail.com (G.A.); gokhanzengin@selcuk.edu.tr (G.Z.); 4 Department of Agricultural Sciences, University of Naples Federico II, 80055 Portici, Italy

**Keywords:** *Lactuca sativa* L., polyphenols, chlorogenic acid, hesperidin, antioxidants, salinity, enzymatic activity, metabolomics, elicitors

## Abstract

The quest for sustainable strategies aimed at increasing the bioactive properties of plant-based foods has grown quickly. In this work, we investigated the impact of exogenously applied phenolics, i.e., chlorogenic acid (CGA), hesperidin (HES), and their combinations (HES + CGA), on *Lactuca sativa* L. grown under normal- and mild-salinity conditions. To this aim, the phenolic profile, antioxidant properties, and enzyme inhibitory activity were determined. The untargeted metabolomics profiling revealed that lettuce treated with CGA under non-stressed conditions exhibited the highest total phenolic content (35.98 mg Eq./g). Lettuce samples grown under salt stress showed lower phenolic contents, except for lettuce treated with HES or HES + CGA, when comparing the same treatment between the two conditions. Furthermore, the antioxidant capacity was investigated through DPPH (2,2-diphenyl-1-picrylhydrazyl), ABTS (2,20-azinobis-(3-ethylbenzothiazoline-6-sulfonate)), and FRAP (ferric reducing antioxidant power) assays, coupled with metal-chelating activity and phosphomolybdenum capacity. An exciting increase in radical scavenging capacity was observed in lettuce treated with exogenous phenolics, in both stress and non-stress conditions. The inhibitory activity of the samples was evaluated against target health-related enzymes, namely cholinesterases (acetylcholinesterase; AChE; butyryl cholinesterase; BChE), tyrosinase, α-amylase, and α-glucosidase. Lettuce treated with HES + CGA under non-stress conditions exhibited the strongest inhibition against AChE and BChE, while the same treatment under salinity conditions resulted in the highest inhibition capacity against α-amylase. Additionally, CGA under non-stress conditions exhibited the best inhibitory effect against tyrosinase. All the functional traits investigated were significantly modulated by exogenous phenolics, salinity, and their combination. In more detail, flavonoids, lignans, and stilbenes were the most affected phenolics, whereas glycosidase enzymes and tyrosinase activity were the most affected among enzyme assays. In conclusion, the exogenous application of phenolics to lettuce represents an effective and green strategy to effectively modulate the phenolic profile, antioxidant activity, and enzyme inhibitory effects in lettuce, deserving future application to produce functional plant-based foods in a sustainable way.

## 1. Introduction

Plant foods are consumed globally as a source of minerals, vitamins, fiber, and other health-promoting compounds, and their consumption is recognized to decrease the risk of metabolic disorders, chronic conditions, and non-communicable diseases [1,2],. Among others, plant-based foods reduce the risk of diabetes, cancer, and cardiovascular diseases [3,4] because, in addition to micro- and macronutrients, they contain many bioactive components such as flavanols, carotenoids, and anthocyanins which have been shown to have health-promoting properties. Phenolic compounds are the most abundant secondary metabolites in plants, accumulating in all tissues and organs, and are at the center of research due to their antioxidant, anti-hypertensive, anti-inflammatory, and anti-carcinogenic effects [5,6]. The increasing awareness of society on the subject has rapidly increased the market demand for fresh products rich in nutritional value and functional properties.

Although the synthesis and accumulation of bioactive compounds in plants are species-specific, they may vary according to growing conditions, soil properties, light, and nutrient range [7]. It is also known that phenolics biosynthesis is induced in response to abiotic stresses, and they are involved in the plant antioxidant defense system [8,9]. Due to the prominent effects of the bioactive molecules in the prevention and control of chronic diseases, the interest towards strategies to increase the phenolic content of plant foods has grown quickly. Besides optimizing environmental conditions, it has been shown that the external application of elicitors can increase the yield and nutritional values of the plants. For example, Grzeszczuk et al. [10] showed that external salicylic acid application to *Salvia coccinea* under salinity reversed the stress effects and increased total polyphenol and total carotenoid contents, compared to untreated plants.

Lettuce (*Lactuca sativa* L.) is an herbaceous crop belonging to the *Asteraceae* family. It is one of the most grown vegetables globally yet is often underestimated in terms of nutritional value. In fact, with its high fiber, mineral, and vitamin (B9, A, C, E, K) content, lettuce is a rich source for diets [11]. Its health benefits also have been associated with high amounts of bioactive compounds such as carotenoids and phenolic acids [12]. In addition to its intestinal regulation and immune system-stimulating properties, bioactive molecules in lettuce reduce the formation of reactive oxygen species and protect cellular components from damage. Furthermore, lettuce leaves are usually consumed raw, which makes the delivery of natural antioxidants to humans more effective than processed vegetables. Recent studies have presented the positive effects of nitrogen supply [13], variety [14], nutritional stress [15], and light [16] on the phenolic accumulation in lettuce. However, to the best of our knowledge, little is known about the changes in the phenolic profile of lettuce under normal and salinity conditions, eventually combined with exogenously applied phenolics. In this regard, the application of phenolics has been reported to alleviate oxidative stress and enhance the antioxidant potential of apple leaves [3] and tomato [17]. Considering that salinity induces redox imbalance in plants, it has been proposed as able to improve quality, including the content of bioactive compounds, in horticultural crops [18]. Interestingly, exogenous phenolics may contribute to shaping functional traits in the crop synergically with salinity while supporting plant growth by mitigating the deleterious effects of salt stress. These phenolics are rather different in terms of chemical structures, and it can be postulated that they can provide different effects in the plant. Moreover, both are common in food byproducts and could be easily recovered at relatively low costs in a circular economy framework. Therefore, this piece of information may represent an effective strategy to modulate the health-promoting potential of lettuce in a rather sustainable manner, paving the way towards the modulation of functional properties in plant-based foods. Therefore, the aim of work is to determine the impact of phenolic applications (the hydroxycinnamic acid chlorogenic acid, the flavanone hesperidin, and their combinations), under salt stress and non-stress conditions, on the phenolic profile and functional traits of lettuce.

## 2. Results

### 2.1. Metabolomic Profiling of Lettuce Treated with Hesperidin and Chlorogenic Acid

The impact of different phenolic compounds, namely hesperidin (HES) and chlorogenic acid (CGA), applied either alone or combined, was evaluated in lettuces grown under salinity and non-salt-stressed conditions. The polyphenolic profile of treated lettuces was evaluated using a metabolomic approach based on ultra-high-pressure liquid chromatography coupled to quadrupole time of flight mass spectrometry (UHPLC-QTOF-MS). The untargeted annotation workflow allowed us to putatively annotate 278 compounds, including 131 flavonoids—i.e., 40 anthocyanins, 25 flavonols, and 66 other flavonoids, and 62 tyrosol equivalents. Additionally, 59 phenolic acids were identified, being mainly composed of hydroxycinnamic acids (42 compounds), together with 19 lignans and 7 stilbenes. The polyphenols annotated are reported in Supplementary Materials (Table S1), comprehensive of their composite mass spectrum (mass and abundance combinations).

After that, to compare the different treatments and conditions, a semi-quantification of polyphenols was carried out for each sample. According to calibration curves from a representative standard compound per class, the results are expressed as mg phenolic equivalents/g dry matter (DM; Table 1).

The lettuce grown under salinity showed a significantly lower recovery of phenolic compound content, particularly in flavonols (*p* < 0.001), lignans, phenolic acids (*p* < 0.01), anthocyanins, and stilbenes (*p* < 0.05). Interestingly, the exogenous application of phenolics featured an increase in stilbenes, lignans and other flavonoids classes—i.e., flavones, flavanones, flavanols, isoflavonoids, and dihydro- chalcones. The phenolics compound applied under standard conditions revealed an exciting increase in low-molecular-weight phenolics, mainly characterized by hydroxy-benzaldehydes or cinnamaldehydes, alkylphenols, and tyrosols, as well as stilbenes, lignans, and other flavonoids (Table 1). Considering the interaction parameters between the condition of growth and phenolic application (S × P), the employment of CGA in combination with HES, under normal conditions, resulted in a significant accumulation of anthocyanins, lignans, and low-molecular-weight phenolics. However, the effect of HES application was shown to have a crucial role in the recovery of other flavonoid classes when salinity conditions were considered.

In detail, the cumulative phenolic content ranged from 25.46 (for HES + CGA, under salinity stress) up to 35.98 (for CGA, under non-stress conditions) mg Eq./g. The presence of salt generally led to lower phenolic content values, except for the treatment with HES. Overall, the samples analyzed revealed a similar phenolic distribution. In this regard, the most abundant classes were represented by low-molecular-weight phenolics (tyrosol derivatives; from 39.42% up to 53.14% of the total phenolic content), lignans (from 17.73% up to 34.19%), and flavonoids (from 17.39% up to 20.89%). Among flavonoids, anthocyanins were the most abundant sub-class (from 65.64% to 74.09% of the total flavonoids). In non-salt conditions, tyrosol equivalents were the most representative class in HES- and HES + CGA-treated lettuces, recording values of 12.09 and 12.51 mg Eq./g, respectively. Additionally, the CGA treatment showed the highest anthocyanins content (4.70 mg Eq./g; *p* < 0.05) when compared to both control and other treatments, and consequently the highest flavonoid content (6.34 mg Eq./g) as well. Indeed, the treatment with CGA led to the highest total phenolic content in lettuce (35.98 mg Eq./g) when considering salinity conditions.

Different results occurred when the salinity stress was considered. Indeed, the lettuce treated only with salt exhibited the highest values of tyrosol equivalents (15.28 mg/g) as well as the highest anthocyanins (4.35 mg/g) and flavonols contents (0.80 mg/g), with the lowest lignans content recorded (5.10 mg/g). In this regard, both treatments with CGA and HES + CGA led to the lowest content of anthocyanins in lettuce (*p* < 0.05), while HES determined the lowest flavonols content (0.27 mg/g) when compared to other treatments under salinity stress and the control sample. Finally, the treatment with HES under salinity was the only one that slightly increased the total phenolic content under salinity stress conditions.

### 2.2. Multivariate Discrimination Analysis Phenolic Profile Following the Different Treatments Applied under Salinity and Non-Salinity Conditions

**Figure 1:**
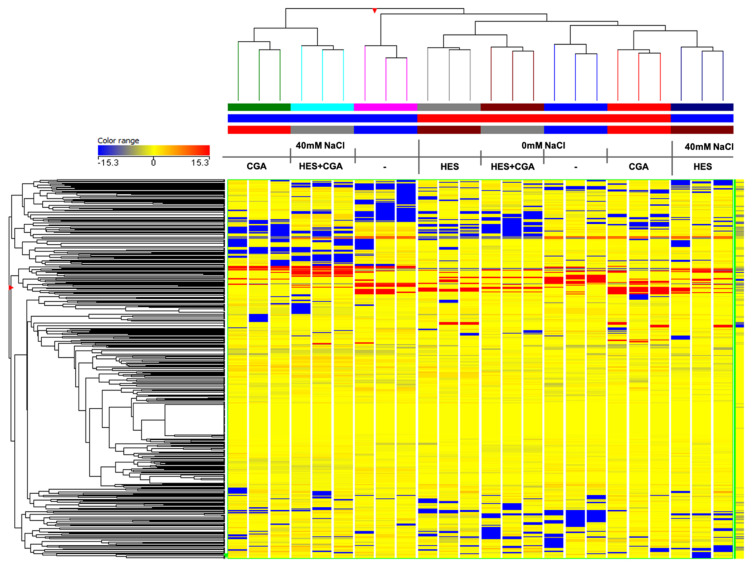
Unsupervised hierarchical clustering analysis (HCA) built using intensity values obtained from UHPLC-QTOF-MS and normalized relative to the median. Lettuces were treated with: 100 µM hesperidin (HES), 50 µM chlorogenic acid (CGA), and 100 µM hesperidin + 50 µM chlorogenic acid (HES + CGA). The treatments were performed both under non-salt (0 mM NaCl) and under salt-stress (40 mM NaCl) conditions.

**Figure 2:**
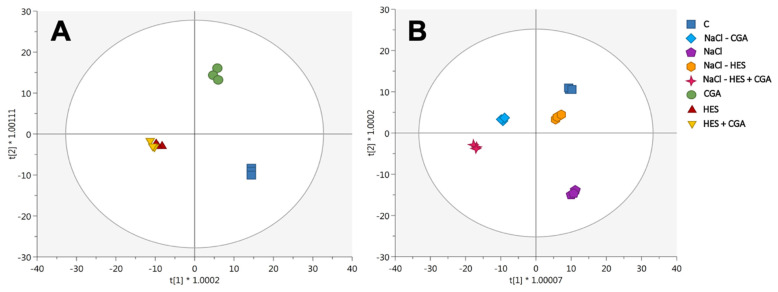
Supervised orthogonal projections to latent structures discriminant analysis (OPLS-DA) score plot built according to the phenolics of lettuces treated with: control non-treated (C), 100 µM hesperidin (HES), 50 µM chlorogenic acid (CGA), and 100 µM hesperidin + 50 µM chlorogenic acid (HES + CGA). The treatments were performed both under non-salt (**A**) and salt-stressed (**B**) conditions.

These results provide phenolic patterns in agreement with both unsupervised hierarchical clustering analysis (HCA) (Figure 1) and supervised orthogonal projection to latent structures discriminant analysis (OPLS-DA) (Figure 2A,B). These approaches have been adopted to find out similarities and dissimilarities across the different treatments. The HCA indicated a strong effect of salinity in modulating the phenolic composition of lettuce, even though the HES treatment clustered separately from the other salt-stress conditions. As shown in Figure 1, two main clusters were generated from the fold-change-based heatmap of HCA. The first cluster consisted of CGA and HES + CGA treatments under salinity conditions. Among the second distinct subcluster, the treatment with HES applied under salt stress was identified with a phenolic profile close to the non-stressed sample group.

Afterward, the supervised OPLS-DA modeling was used to extrapolate the phenolic compounds, allowing the discrimination between different treatments, and identifying their modulation according to saline conditions. The first OPLS-DA score plot (Figure 2A) considered only the impact of different treatments on the phytochemical profile. As shown in Figure 2A, the first latent vector led to a clear separation between treatments, highlighting the greater similarity between HES and HES + CGA treatments. The outcome was in line with the trend already noted in the HCA. The parameters related to the goodness of fit and the prediction capacity of the model were determined, being goodness-of-fit (R^2^Y cum) = 0.99 and goodness-of-prediction (Q^2^ cum) = 0.87. Afterwards, a Variable Importance in Projection (VIP) approach was exploited to identify those compounds mostly affected by the treatments. To this aim, we have retained those phenolic compounds possessing a VIP score > 1.2 and recording majorly in flavonoids and phenolic acids with a score > 1.3. However, the compound recording the higher value was resveratrol 5-O-glucoside (VIP score > 1.4), belonging to the class of stilbenes (Table S1). In addition, 3,4-dicaffeoylquinic acid was the compound with the highest up-accumulation value considering the pairwise comparison between CGA (logFC = 18.59) and HES (logFC = 20.13) vs. control, while a slight down-accumulation (log FC = −0.01) was found for HES + CGA vs. control. Additionally, vitisin A demonstrated an up-accumulation for all treatments when compared to the control, of logFC = 19.33 (CGA vs. control), logFC = 18.84 (HES vs. control), and logFC = 19.64 (HES + CGA vs. control).

Finally, in the second OPLS-DA score plot (Figure 2B), a clear separation between control and stress-treated samples was obtained based on the phenolic profile, except for the HES, which demonstrated a profile more similar to the control. Additionally, the model parameters were quite good; R^2^Y cum = 0.99 and Q^2^ cum = 0.87. The most discriminant compounds outlined by the VIP selection method were phenolic acids and flavonoids. Notably, kaempferide (flavonols), 6″-O-malonylglycitin (isoflavonoids), and p-coumaroyl glucose (hydroxycinnamic acids) showed a discriminant VIP score > 1.3, followed by quercetin, malvidin galactosides, and 3,4-diferuloylquinic acid (hydroxycinnamic acids) with a VIP score > 1.28. Interestingly, the vitisin A (VIP score = 1.32) was the most up-accumulated phenolic marker detected in the salt-treated samples (compared to the control), while the spinacetin 3-*O*-glucosyl-(1-6)-glucoside was the most down-accumulated.

### 2.3. Antioxidant Capacity

The in vitro antioxidant assays were carried out to investigate radical scavenger properties of lettuce treated with exogenous phenolic compounds, considering two different growth conditions (stress and non-stress). Notably, several complementary assays were performed to obtain a broad spectrum of information able to enhance the obtained result. In this regard, DPPH (2,2-diphenyl-1-picrylhydrazyl), ABTS (2,20-azinobis-(3-ethylbenzothiazoline-6-sulfonate)), and FRAP (ferric reducing antioxidant power) assays, coupled with metal-chelating activity assay and phosphomolybdenum assay, have been performed to find out differences in the antioxidant capacity of lettuces due to different treatments, and the results are shown in Table 2.

Overall, the effect of salinity on lettuce decreased the antioxidant capacity significantly, considering ABTS, FRAP assays, and metal-chelating properties (*p* < 0.001). However, the exogenous application of phenolics increased the antioxidant capacity for all assays carried out compared to control non-treated. Considering the interaction between growth parameters and phenolic application, treatments with HES and HES + CGA under standard growth conditions improved the antioxidant capacity of lettuces (Table 2). Particularly, a significant increase in FRAP capacity was reported, recording values from 15.72 to 15.81 TE/g, followed by ABTS values ranging from 8.62 to 9.50 TE/g, and phosphomolybdenum capacity ranging from 0.85 to 0.94 mmol TE/g. The metal-chelating properties of lettuces were also increased to 32.71–33.02 EDTAE/g.

Notably, the treatments with HES applied on lettuces grown under salinity resulted in the highest ABTS-scavenging ability (10.56 mg TE/g), phosphomolybdenum capacity (1.09 mmol TE/g), and metal-chelating potential (34.24 mg EDTAE/g), suggesting the effect of treatments in improving the radical scavenger capacity of lettuce under salt stress. Moreover, the application of HES + CGA under salinity recorded a great value of FRAP capacity, of 14.52 mg TE/g, compared to lettuces grown under salinity alone (13.89 mg TE/g).

### 2.4. Enzyme Inhibition Activity

The enzyme inhibition activity of methanolic extracts obtained from lettuces treated with HES, CGA, and HES + CGA under stress and non-stress conditions was investigated. Cholinesterases (acetylcholinesterase—AChE; butyrylcholinesterase—BChE), tyrosinase, α-amylase, and α-glucosidase were selected as target enzymes. The results are shown in Table 3. In general, the effect of salinity on lettuces resulted in a significant decrease in the inhibitory capacity of the enzymes. However, the employment of exogenous phenolics as treatments produces a significant increase in this activity, with an efficiency scale represented by HES + CGA, CGA and HES.

Considering AChE and BChE inhibition capacity, the lettuce extract treated with HES + CGA (under non-stress conditions) exhibited the strongest inhibition properties, being 2.34 and 2.83 mg GALAE/g, respectively. Despite the salt treatment leading to decreased inhibition capacity on both AChE and BChE enzymes, the treatment with HES + CGA and CGA increased the enzyme inhibition capacity compared to lettuces grown under salinity alone. Regarding the tyrosinase enzyme inhibition, CGA (under non-stress condition) exhibited the best inhibitory effect with the value of 50.56 mg KAE/g. The same treatment was recorded as the best candidate under salinity conditions, equal to HES + CGA (48.74 and 49.69 mg KAE/g, respectively). Generally, treatments containing CGA, under stress and non-stress conditions, have shown a positive inhibition capacity against tyrosinase. The α-amylase inhibition assay resulted in a similar range in all tested groups (0.33–0.41 mmol ACAE/g), showing the treatment with HES + CGA as having the highest inhibition capacity under stress conditions. Similarly, the α-glucosidase inhibitory capacity resulted in the same trends among treatments (ranging from 0.37 up to 1.06 mmol ACAE/g). From an overall perspective, the treatments with CGA and HES (alone or combination) under stress or non-stress conditions positively contributed to lettuce samples’ observed enzyme inhibition capacity.

## 3. Discussion

Lettuce (*Lactuca sativa* L.) is known as an important source of phytochemicals such as fibers, minerals, vitamins, and phenolic compounds, having potential health benefits and antioxidant properties. In our experimental conditions, the untargeted metabolomics approach pointed out a broad and diverse presence of flavonoids, tyrosol derivatives (other polyphenols), and phenolic acids. According to previous works [19,20], caffeic, coumaric, and ferulic acids and their esters and glucosides (hydroxycinnamic acids), along with the isomers of dihydroxybenzoic, syringic, and vanillic acid, as well as galloyl glucose (hydroxybenzoic acids), were found to be the most representative phenolic metabolites in lettuce. Additionally, quercetin and luteolin and their glucosides, scutellarein, naringenin, and hesperetin were the most representative flavonoids detected from our findings. Notably, the application of exogenous CGA increased the total phenolic composition, corroborating the results of a study by Yimeng Mei et al. [3]. These results indicate an enhancement of the radical scavenging activity and reducing power, as well as of the antioxidant activities related to the application of chlorogenic acid to apple leaves. Furthermore, another study by El-Shafey and AbdElgawad [21] demonstrated that exogenous luteolin compounds alleviate the detrimental effects of salinity in maize, enhancing oxidative stress tolerance in plants. Accordingly, we detected an increased total antioxidant capacity (as phosphomolybdenum assay) in salt-stressed and phenol-treated lettuces, confirming the elicitation capacity of exogenous phenolics in alleviating oxidative stress caused by salinity. Therefore, the application of exogenous antioxidant compounds to plants can modulate and elicitate their functional properties. In this regard, no previous studies have been conducted on lettuce, according to our knowledge.

Anthocyanins, flavonols, and other flavonoids were the three phenolic classes showing the most significant modulation in response to the interaction between salinity and application of exogenous phenolics. All these classes belong to flavonoids, a class of phenolics synthesized via the phenylpropanoid pathway from the condensation of phenylalanine and 4-coumaroyl-CoA. This upstream step, catalyzed by chalcone synthase, produces scaffolds for all flavonoids that belong to the different flavonoid subclasses. Therefore, their joint involvement in response to exogenous phenolics is not surprising. However, the following phenolic class, being involved in salinity x phenolic treatments, was the class of lignans. These phenolics originate from coniferyl alcohol, and thus they arise from a rather unrelated biosynthetic pathway.

Moreover, the phenolic content is significantly influenced by cultivars, environmental conditions, and other sources of stress, inducing plants to activate several defense mechanisms. Indeed, secondary metabolism varies between species and reflects their adaptation [22]. Generally, salinity leads to the accumulation of antioxidant compounds that play a crucial role in counterbalancing oxidative stress [23]. However, in our results, the antioxidant capacity decreased significantly in the presence of salinity, denoting an insufficient production of antioxidant compounds. Indeed, the total phenolic content in salt-stressed lettuces decreased when compared to the control. Considering the treatment with exogenous phenolics (particularly with HES), their application resulted in a significant accumulation of endogenous phenolic compounds and enhancement of antioxidant activity (both in stress and non-stress conditions). These results agree with previous works, in which the total phenolic content was reduced in the presence of salt [24,25]. This could be due to alterations in the metabolism of phenolic compounds following treatments with salt. In fact, [26] demonstrated that lettuce plants treated with 100 mM NaCl exhibited the lowest activities of some enzymes involved in the biosynthetic pathway of phenolic compounds (i.e., shikimate dehydrogenase, phenylalanine ammonia-lyase, and 4-coumarate coenzyme A ligase), and the maximum activity of the enzyme polyphenol oxidase, which on the contrary is involved in phenol oxidation processes. Notwithstanding, plants’ responses are contrasting and might be explained by differences between species, which present different salt sensitivity, but could also be related to the use of varying times of stress exposure, modes of cultivation, and combinations of stress factors (i.e., osmotic, drought, and nutritional effects) [22].

Enzymes are important targets for managing global health problems, including diabetes mellitus, Alzheimer’s, and obesity. The inhibition of enzymes might alleviate the symptoms observed in the pathologies of these diseases [27]. Several compounds have been synthetically produced and sold as inhibitors by the pharmaceutical and food industries in this sense. However, most of these synthetics have unpleasant side effects on human health and need to be changed with natural ones, which are safer and more effective. In this regard, natural sources of enzyme inhibitors have a great interest in the scientific platform [28,29,30]. In the current study, the enzyme inhibitory effects of lettuce samples under different growth conditions were investigated. As shown in Table 3, the phenolics application under stress and non-stress conditions contributed to the observed enzyme-inhibitory properties. For example, in non-stress groups, the combination of HES + CGA was 40% more active on AChE than the control. In addition, the application of HES and CGA was more active on AChE compared with the control, by 28% and 26%, respectively. This effect could be explained by the synergetic effects of these two phenolic compounds on enhancing plant metabolic response to cope with abiotic stresses. Indeed, some authors reported the synergistic effects among phenolic compounds on biological activity improvement, including antioxidant and enzyme inhibition effects [31,32,33,34,35]. In stressed conditions, CGA and HES application combined improved the enzyme inhibition effects compared with the salt group (except for BChE). In addition, α-glucosidase inhibitory was not much affected by the treatment of phenolics (as alone and combination) under tested conditions. To the best of our knowledge, scientific data regarding the impact of exogenous phenolic treatments on the enzyme-inhibitory effects of lettuce is scarce. In our earlier study [36], we reported the enzyme inhibitory properties of lettuce grown in hydroponic conditions in a genotype-dependent manner. From this point, the presented results could highlight effective scientific strategies for yielding more functional lettuce samples in the future.

## 4. Materials and Methods

### 4.1. Plant Material, Growth Conditions, and Treatment

Lettuce (*Lactuca sativa* L., cultivar) seeds were surface sterilized in sodium hypochlorite solution (5% NaOCl) for 10 min and rinsed thoroughly. After being soaked in distilled water overnight, seeds were allowed to germinate on wet filter paper. The one-week-old seedlings were transferred to ½ Hoagland solution under controlled conditions (16 h light/8 h dark regime; 24 °C; 70% relative humidity; 350 µmol m^−2^ s^−2^ photon flux density). The hydroponic medium was refreshed every other day during the 21-day growth period. Since there is no study on the exogenous application of hesperidin or chlorogenic acid to lettuce plants, pre-trial groups between 25 and 200 µM were established using the limited data in the literature [3,37]. At the end of the 10-day treatment, the appropriate concentrations were determined as 50 µM chlorogenic acid and 100 µM hesperidin by comparing the groups’ growth parameters and biomass accumulations. Salt stress was chosen as 40 mM NaCl for the stress treatment following previous studies [38,39]. The experimental design and treatment groups are summarized in Table 4. Plants were treated with hesperidin, chlorogenic acid, and NaCl dissolved in a half-strength Hoagland solution at the decided concentrations. After 10 days of treatment, the plants were harvested for further analysis.

### 4.2. Untargeted Phenolic Compounds Profiling by UHPLC-QTOF Mass Spectrometry

Lettuce was extracted by using a homogenizer-assisted extraction (Ultra-Turrax; Polytron PT, Switzerland) in a hydroalcoholic solution (80:20 methanol: water), acidified with 0.1% (*v*/*v*) formic acid. Then, the samples were centrifuged for 10 min at 6000× *g*, in a refrigerated (4 °C) centrifuge, and the supernatants filtered using 0.22 µm cellulose syringe filters and transferred into UHPLC vials. A quadrupole-time-of-flight instrument (Agilent 6550 iFunnel), coupled to an ultra-high-pressure liquid chromatographic system (Agilent 1200 series), was used for the untargeted profiling. The UHPLC-QTOF-MS analytical conditions are described in previous works [40]. In brief, the separation was performed under a water–acetonitrile gradient elution starting from 6% acetonitrile to 94% in 33 min. The column employed was an Agilent Zorbax Eclipse Plus C18 column (50 × 2.1 mm, 1.8 μm), the injection volume was 6 μL, and each sample was analyzed in triplicate. The mass spectrometer worked in SCAN mode with a range of 100 to 1200 m/z, positive polarity and extended dynamic range mode, with a nominal mass resolution of 30,000 FWHM.

The raw data were then processed using the Profinder B.07 software (Agilent Technologies), according to the “find-by-formula” algorithm. Monoisotopic accurate mass was used considering the entire isotopic profile, the combination of monoisotopic mass, isotopes ratio, and spacing to reach level 2 of confidence for identification [41]. The database exported from Phenol-Explorer 3.6 was used as a reference for identification considering 5 ppm for mass accuracy, following mass (5 ppm accuracy) and retention time (0.05 min maximum shift) alignments.

### 4.3. In Vitro Antioxidant Assays

Antioxidant capacity was evaluated by different chemical assays, including quenching free radicals (DPPH and ABTS), reducing power (FRAP), metal chelating, and phosphomolybdenum. The experimental procedures of the assays were given in our earlier paper [42]. Standard compounds (Trolox and EDTA (for metal chelating)) were used to express the antioxidant effects (mg standard equivalent/g dry extract)

### 4.4. In Vitro Enzyme-Inhibitory Assays

Enzyme-inhibitory assays (acetylcholinesterase (AChE), butyrylcholinesterase (BChE), tyrosinase, α-amylase, α-glucosidase) were performed according to previously described methods [43]. Standard compounds, galantamine (for cholinesterases), kojic acid (for tyrosinase) and acarbose (for α-amylase and α-glucosidase), were used to express the enzyme-inhibitory potential (mg standard equivalent/g dry extract).

### 4.5. Statistical Analysis

Considering data from each assay, a one-way analysis of the variance (ANOVA) was performed by using the software PASW Statistics 26.0 (SPSS Inc., Chicago, IL, USA) followed by Duncan’s post hoc test (*p* < 0.05). Moreover, the software Mass Profiler Professional 12.6 (Agilent Technologies) was used for data elaboration, as previously reported [44,45]. Afterwards, the unsupervised hierarchical clustering (HCA) and the supervised orthogonal partial least squares discriminant analysis (OPLS-DA) were carried out. The unsupervised hierarchical cluster analysis was built according to Euclidean distance and Ward’s linkage, to underline similarities and differences across the treatments. In addition, the OPLS-DA was carried out using SIMCA 13 software (Umetrics, Malmo, Sweden) and the model cross-validated and inspected for outliers. Those parameters related to the goodness-of-fit R2Y and goodness-of-prediction Q2Y were recorded. The Variable Importance in Projection (VIP) selection method was depicted to select those metabolites having the highest discriminant potential [46]. In particular, we considered those metabolites possessing the highest degree in discrimination (VIP score >1.2).

## 5. Conclusions

In this work, we evaluated the impact of phenolics exogenously applied to *Lactuca sativa* L. grown under normal and salinity conditions on the phenolic profile and the enzyme-inhibitory activity. Quantitative differences were seen, as provided by multivariate discrimination analysis (i.e., HCA and OPLS-DA), thus highlighting a phenolic-specific and distinct effect for CGA and HES. Lettuce treated with CGA under non-stressed conditions exhibited the highest total phenolic content. Notably, when comparing the same treatment among the two conditions (i.e., non-stressed vs. salt-stressed), lettuce samples grown under salinity showed lower phenolic contents, with the sole exception of lettuce treated with HES or HES + CGA. Indeed, the treatment with HES under salinity exhibited a slightly higher phenolic content than that treated with HES but in non-stressed conditions. The same treatments were also related to a higher antioxidant capacity of the samples, considering ABTS, FRAP, metal-chelating, and phosphomolybdenum capacities. Furthermore, we investigated the enzyme inhibition activity of lettuce extracts against target enzymes, namely cholinesterases (acetyl cholinesterase; AChE; butyryl cholinesterase; BChE), tyrosinase, α-amylase, and α-glucosidase. Lettuce treated with HES + CGA under non-stress conditions exhibited the strongest inhibition against AChE and BChE, while the same treatment under salinity conditions resulted in the highest inhibition capacity against α-amylase. Additionally, CGA under non-stress conditions exhibited the best inhibitory effect against tyrosinase. On the contrary, no significant differences were seen in β-glucosidase inhibition among different treatments and conditions. In conclusion, we demonstrated that the exogenous application of phenolics to lettuce effectively modulates the phenolic profile and the enzyme inhibitory effects, both in normal and salinity conditions. Based on our results, the application of antioxidant compounds to plants could represent a valuable strategy to produce food with tailored functional features. Furthermore, these phenolic compounds are highly accessible in nature from multiple sources and at low prices. Interestingly, many raw materials, such as waste streams from food production, were shown in different studies to be a relevant source for bioactive metabolites. Indeed, a future strategy could be to develop green and sustainable extraction methods from different waste/byproduct matrices, with the aim of recovering plant-active antioxidant compounds.

## Figures and Tables

**Table 1:** Semi-quantitative analysis of methanolic extracts obtained from lettuces treated with: standard non-treated, 100 µM hesperidin (HES), 50 µM chlorogenic acid (CGA), and 100 µM hesperidin + 50 µM chlorogenic acid (HES + CGA). The treatments were performed both under mild salinity (40 mM NaCl) and non-salt-stress conditions.

Source of Variance	Anthocyanins	Other Flavonoids	Flavonols	Lignans	Other Polyphenols	Phenolic Acids	Stilbenes
	mg Eq. g^−1^ DM	mg Eq. g^−1^ DM	mg Eq. g^−1^ DM	mg Eq. g^−1^ DM	mg Eq. g^−1^ DM	mg Eq. g^−1^ DM	mg Eq. g^−1^ DM
**Salinity (S)**							
Standard	4.05 ± 0.13 a	1.08 ± 0.05	0.59 ± 0.07 a	8.88 ± 0.46 a	14.44 ± 1.07	1.97 ± 0.06 a	0.99 ± 0.18 b
Saline	3.85 ± 0.16 b	1.15 ± 0.09	0.51 ± 0.06 b	6.81 ± 0.58 b	12.85 ± 0.78	1.71 ± 0.07 b	1.45 ± 0.27 a
**Phenolic application (P)**							
No application	4.12 ± 0.12 a	0.96 ± 0.07 c	0.78 ± 0.02 a	7.32 ± 1.07	14.86 ± 0.98	1.92 ± 0.08	0.58 ± 0.02 b
HES	3.99 ± 0.15 a	1.36 ± 0.06 a	0.24 ± 0.01 d	7.80 ± 0.84	13.22 ± 0.98	1.92 ± 0.10	1.27 ± 0.31 a
CGA	4.18 ± 0.23 a	1.15 ± 0.12 b	0.63 ± 0.05 b	7.50 ± 0.37	15.17 ± 1.94	1.87 ± 0.13	1.22 ± 0.24 a
HES + CGA	3.53 ± 0.20 b	0.99 ± 0.07 c	0.54 ± 0.05 c	8.76 ± 1.01	11.32 ± 0.79	1.66 ± 0.07	1.81 ± 0.45 a
**S × P**							
Standard × No application	3.89 ± 0.15 cd	1.06 ± 0.08 bcd	0.76 ± 0.02 a	9.54 ± 0.15 ab	14.45 ± 1.79 abc	2.02 ± 0.05	0.61 ± 0.03 c
Standard × HES	3.71 ± 0.12 d	1.25 ± 0.06 ab	0.22 ± 0.01 e	7.04 ± 0.03 bcd	12.09 ± 1.87 bc	2.02 ± 0.12	1.64 ± 0.59 b
Standard × CGA	4.70 ± 0.07 a	0.90 ± 0.09 cd	0.74 ± 0.02 a	8.10 ± 0.22 bc	18.70 ± 1.82 a	2.05 ± 0.20	0.80 ± 0.11 bc
Standard × HES + CGA	3.92 ± 0.14 cd	1.12 ± 0.07 bc	0.65 ± 0.02 b	10.85 ± 0.67 a	12.51 ± 1.27 bc	1.80 ± 0.09	0.89 ± 0.24 bc
Saline × No application	4.35 ± 0.05 ab	0.86 ± 0.08 d	0.80 ± 0.03 a	5.10 ± 0.85 d	15.28 ± 1.21 ab	1.82 ± 0.14	0.55 ± 0.03 c
Saline × HES	4.27 ± 0.16 bc	1.47 ± 0.06 a	0.27 ± 0.01 e	8.56 ± 1.73 abc	14.35 ± 0.18 abc	1.83 ± 0.17	0.90 ± 0.03 bc
Saline × CGA	3.67 ± 0.07 d	1.40 ± 0.07 a	0.52 ± 0.02 c	6.91 ± 0.55 cd	11.64 ± 1.77 bc	1.69 ± 0.12	1.64 ± 0.31 b
Saline × HES + CGA	3.14 ± 0.18 e	0.86 ± 0.06 d	0.44 ± 0.01 d	6.67 ± 0.55 cd	10.13 ± 0.30 c	1.52 ± 0.03	2.72 ± 0.35 a
**Significance**							
S	*	ns	***	**	ns	**	*
P	***	***	***	ns	ns	ns	**
S × P	***	***	***	**	*	ns	**

Data are mean ± standard error; n = 3. The symbols ns, *, **, and *** indicate a nonsignificant or a significant statistical difference at *p* < 0.05, 0.01, and 0.001, respectively. Different letters within each column indicate significant differences according to Duncan’s multiple range test (*p* < 0.05). The Salinity factor was compared with the independent Student’s t-test.

**Table 2:** Antioxidant capacity of methanolic extracts obtained from lettuces treated with: standard non-treated, 100 µM hesperidin (HES), 50 µM chlorogenic acid (CGA), and 100 µM hesperidin + 50 µM chlorogenic acid (HES + CGA). The treatments were performed both under mild salinity (40 mM NaCl) and non-salt-stress conditions.

Source of Variance	DPPH	ABTS	FRAP	Metal Chelating	Phosphomolybdenum
	mgTE g^−1^	mgTE g^−1^	mgTE g^−1^	mg EDTAE g^−1^	mmol TE g^−1^
**Salinity (S)**					
Standard	1.17 ± 0.13 b	8.86 ± 0.14 a	15.37 ± 0.14 a	30.50 ± 0.78 a	0.87 ± 0.02
Saline	2.49 ± 0.34 a	8.10 ± 0.47 b	12.71 ± 0.53 b	26.46 ± 1.38 b	0.90 ± 0.04
**Phenolic application (P)**					
No application	0.94 ± 0.00 b	8.12 ± 0.38 b	14.59 ± 0.31 b	25.94 ± 1.58 c	0.83 ± 0.03 b
HES	2.54 ± 0.56 a	9.59 ± 0.44 a	12.93 ± 1.26 c	28.55 ± 1.86 b	0.97 ± 0.06 a
CGA	2.24 ± 0.46 a	8.22 ± 0.15 b	13.50 ± 0.54 c	30.51 ± 1.68 a	0.84 ± 0.02 b
HES + CGA	1.48 ± 0.31 b	7.99 ± 0.68 b	15.16 ± 0.41 a	28.90 ± 1.85 b	0.90 ± 0.02 ab
**S × P**					
Standard × No application	nd	8.89 ± 0.27 c	15.28 ± 0.07 ab	29.47 ± 0.10 c	0.86 ± 0.03 bc
Standard × HES	1.39 ± 0.27	8.62 ± 0.09 c	15.72 ± 0.07 a	32.71 ± 0.18 b	0.85 ± 0.05 bc
Standard × CGA	1.25 ± 0.20	8.42 ± 0.09 cd	14.68 ± 0.09 bc	26.77 ± 0.27 d	0.84 ± 0.01 bc
Standard × HES + CGA	0.88 ± 0.08	9.50 ± 0.09 b	15.81 ± 0.19 a	33.02 ± 0.36 b	0.94 ± 0.01 b
Saline × No application	0.94 ± 0.00	7.35 ± 0.23 e	13.89 ± 0.01 c	22.40 ± 0.07 f	0.81 ± 0.07 c
Saline × HES	3.68 ± 0.40	10.56 ± 0.08 a	10.13 ± 0.30 e	24.40 ± 0.09 e	1.09 ± 0.03 a
Saline × CGA	3.24 ± 0.10	8.03 ± 0.24 d	12.31 ± 0.18 d	34.24 ± 0.13 a	0.85 ± 0.05 bc
Saline × HES + CGA	2.08 ± 0.34	6.48 ± 0.08 f	14.52 ± 0.63 bc	24.79 ± 0.01 e	0.85 ± 0.01 bc
**Significance**					
S	***	***	***	***	ns
P	***	***	***	***	**
S × P	ns	***	***	***	**

Data are mean ± standard error; n = 3. The symbols ns, *, **, *** indicate nonsignificant or significant at *p* < 0.05, 0.01, and 0.001, respectively. Different letters within each column indicate significant differences according to Duncan’s multiple range test (*p* < 0.05). The Salinity factor was compared with the independent Student’s *t*-test. “nd” denotes non-detectable values.

**Table 3:** Enzyme inhibitory capacity of methanolic extracts obtained from lettuces treated with: standard non-treated, 100 µM hesperidin (HES), 50 µM chlorogenic acid (CGA), and 100 µM hesperidin + 50 µM chlorogenic acid (HES + CGA). The treatments were performed both under salt (NaCl) and non-salt stress conditions.

Source of Variance	AChE	BChE	Tyrosinase	α-Amylase	α-Glucosidase
	mg GALAE g^−1^	mg GALAE g^−1^	mg KAE g^−1^	mmol ACAE g^−1^	mmol ACAE
**Salinity (S)**					
Standard	2.06 ± 0.08 a	2.48 ± 0.09 a	46.80 ± 0.78	0.36 ± 0.01 b	1.05 ± 0.01 a
Saline	1.93 ± 0.06 b	1.81 ± 0.10 b	47.59 ± 0.57	0.39 ± 0.01 a	0.83 ± 0.09 b
**Phenolic application (P)**					
No application	1.79 ± 0.08 c	2.20 ± 0.12	46.16 ± 0.69 c	0.38 ± 0.01 a	1.05 ± 0.01 a
HES	1.89 ± 0.12 c	2.19 ± 0.18	45.17 ± 0.65 c	0.36 ± 0.01 b	0.71 ± 0.16 b
CGA	2.05 ± 0.04 b	1.95 ± 0.21	49.65 ± 0.49 a	0.37 ± 0.01 b	0.97 ± 0.04 a
HES + CGA	2.26 ± 0.06 a	2.25 ± 0.26	47.79 ± 0.88 b	0.39 ± 0.01 a	1.02 ± 0.02 a
**S × P**					
Standard × No application	1.67 ± 0.12 d	2.26 ± 0.12 bc	46.81 ± 0.88 b	0.38 ± 0.01 bcd	1.04 ± 0.01 a
Standard × HES	2.13 ± 0.05 abc	2.54 ± 0.21 ab	43.93 ± 0.72 c	0.33 ± 0.01 f	1.06 ± 0.01 a
Standard × CGA	2.11 ± 0.05 abc	2.30 ± 0.06 bc	50.56 ± 0.51 a	0.35 ± 0.01 e	1.03 ± 0.01 a
Standard × HES + CGA	2.34 ± 0.10 a	2.83 ± 0.10 a	45.88 ± 0.39 bc	0.37 ± 0.01 d	1.06 ± 0.01 a
Saline × No application	1.91 ± 0.02 c	2.14 ± 0.23 bcd	45.51 ± 1.08 bc	0.38 ± 0.01 cd	1.06 ± 0.01 a
Saline × HES	1.66 ± 0.10 d	1.85 ± 0.05 cde	46.42 ± 0.20 b	0.39 ± 0.01 b	0.37 ± 0.10 b
Saline × CGA	1.99 ± 0.03 bc	1.59 ± 0.29 de	48.74 ± 0.34 a	0.39 ± 0.01 bc	0.91 ± 0.07 a
Saline × HES + CGA	2.17 ± 0.02 ab	1.67 ± 0.03 e	49.69 ± 0.35 a	0.41 ± 0.01 a	0.99 ± 0.03 a
**Significance**					
S	*	***	ns	***	***
P	***	ns	***	***	***
S × P	**	*	***	***	***

Values are reported as mean ± standard deviation of three parallel experiments. The symbols ns, *, **, *** indicate non-significant or significant at *p* < 0.05, 0.01, and 0.001, respectively. Different letters within each column indicate significant differences according to Duncan’s multiple-range test (*p* < 0.05). The Salinity factor was compared with the independent Student’s *t*-test. GALAE: galantamine equivalent; KAE: kojic acid equivalent; ACAE: acarbose equivalent.

**Table 4:** The experimental design and plant growth conditions.

Groups	Treatments
Control	
Hesperidin	100 µM hesperidin
Chlorogenic acid	50 µM chlorogenic acid
Hesperidin + Chlorogenic acid	100 µM hesperidin + 50 µM chlorogenic acid
Salinity	40 mM NaCl
Salinity—Hesperidin	40 mM NaCl + 100 µM HES
Salinity—Chlorogenic acid	40 mM NaCl + 50 µM CGA
Salinity—Hesperidin + Chlorogenic acid	40 mM NaCl + 100 µM HES + 50 µM CGA

Abbreviation: sodium chloride (NaCl); hesperidin (HES); chlorogenic acid (CGA).

## Data Availability

The whole dataset is enclosed in this paper as supplementary material.

## Supplementary Materials

The following are available online at https://www.mdpi.com/article/10.3390/plants10071457/s1, Table S1: sheet1—Dataset, sheet2—VIP markers Model A, sheet3—VIP markers Model B.
